# Characterization of the RNase R association with ribosomes

**DOI:** 10.1186/1471-2180-14-34

**Published:** 2014-02-11

**Authors:** Michal Malecki, Cátia Bárria, Cecilia M Arraiano

**Affiliations:** 1 Instituto de Tecnologia Química e Biológica (ITQB), Oeiras, Portugal; 2 Institute of Genetics and Biotechnology, Faculty of Biology, University of Warsaw, Warsaw, Poland

**Keywords:** Ribonucleases, RNA, Ribosomes, RNase R

## Abstract

**Background:**

In this study we employed the TAP tag purification method coupled with mass spectrometry analysis to identify proteins that co-purify with *Escherichia coli* RNase R during exponential growth and after temperature downshift.

**Results:**

Our initial results suggested that RNase R can interact with bacterial ribosomes. We subsequently confirmed this result using sucrose gradient ribosome profiling joined with western blot analysis. We found that RNase R co-migrates with the single 30S ribosomal subunits. Independent data involving RNase R in the rRNA quality control process allowed us to hypothesize that the RNase R connection with ribosomes has an important physiological role.

**Conclusions:**

This study leads us to conclude that RNase R can interact with ribosomal proteins and that this interaction may be a result of this enzyme involvement in the ribosome quality control.

## Background

RNase R is an important *Escherichia coli* exonuclease [1,2]. The enzyme is distinguished from other exoribonucleases by the ability to degrade RNA secondary structures without the aid of a helicase activity [3-5]. It is able to degrade these secondary structures only in the presence of a 3′ single-stranded overhang to which it can bind and initiate degradation. The structure of this protein remains unknown and most of the knowledge on RNase R structure is based on the available structures of RNase II and Rrp44. RNase R has a RNB catalytic domain flanked by RNA binding domains: CSD1 and CSD2 located at the N-terminus and a C-terminal S1 domain, following the typical modular organization on RNB family of enzymes. RNase R was shown to be involved in several cellular processes. It is a cold induced protein suggesting its involvement in bacterial adaptation to low temperatures [6]. Its importance for RNA metabolism in the cold relies on the ability to remove highly structured RNAs that are stabilized under these conditions [7]. RNase R takes part in the degradation of mRNAs, and is especially important in the removal of mRNAs with stable stem loops such as REP elements [8]. *In vitro* this enzyme is able to digest highly structured RNAs like rRNA suggesting that RNase R is involved in the removal of these molecules *in vivo*[4]. Some helicase activity independent on exonuclease activity was shown for RNase R [5]. Moreover, RNase R in concert with PNPase was shown to be involved in rRNA quality control [9]. Recent studies show that RNase R is involved in ribosome quality control and degradation, working together with the newly discovered endonuclease YbeY [10].

In stationary phase or upon drop of the temperature, RNase R transcript and protein are considerably stabilized. Due to its stabilization, RNase R levels increase dramatically with an increase of about 10 fold upon a temperature downshift and about 2 fold in stationary phase [6]. Protein stability changes rely on the specific acetylation of the C-terminal Lys544 residue. Acetylation of the Lys544 residue regulates the tmRNA and SmpB binding to the C-terminal region of RNase R [11]. In stationary phase the acetylating enzyme is absent. As a consequence tmRNA and SmpB bind poorly to the C-terminal region of RNase R and the enzyme is stable [11].

Large-scale analysis of protein complexes in *E. coli* growing under exponential phase did not detect strong interactions between RNase R and other proteins [12]. However, immunoprecipitation studies suggest that RNase R may interact with other proteins such as the components of tmRNA machinery [13].

In this study we employed the TAP tag purification method together with mass spectrometry to identify the proteins that co-purify with RNase R after a temperature downshift and in exponentially growing cells (See Additional file 1). Despite not having identified any stable complexes, our RNase R purifications were enriched with ribosomal proteins. This enrichment was still observed after RNase A treatment suggesting that RNase R could be bound to the ribosome. At least for rRNA degradation, it was shown that PNPase works in concert with RNase R in the ribosome quality control process and only the deletion of both proteins gives a lethal phenotype characterized by the accumulation of undegraded, deficient ribosomal subunits [9]. Moreover, while this manuscript was in review an independent laboratory came out with similar evidences using different approaches [14]. Our results using sucrose polysome gradients combined with western blot technique demonstrated that *in vivo* most of the RNase R signal is connected with the 30S ribosomal subunit. All of these results, together with reports on the involvement of RNase R in ribosome quality control, show that RNase R interaction with the ribosomes may be an important physiological phenomenon.

## Results

### Preparation of RNase R-TAP strain

**Figure 1:**
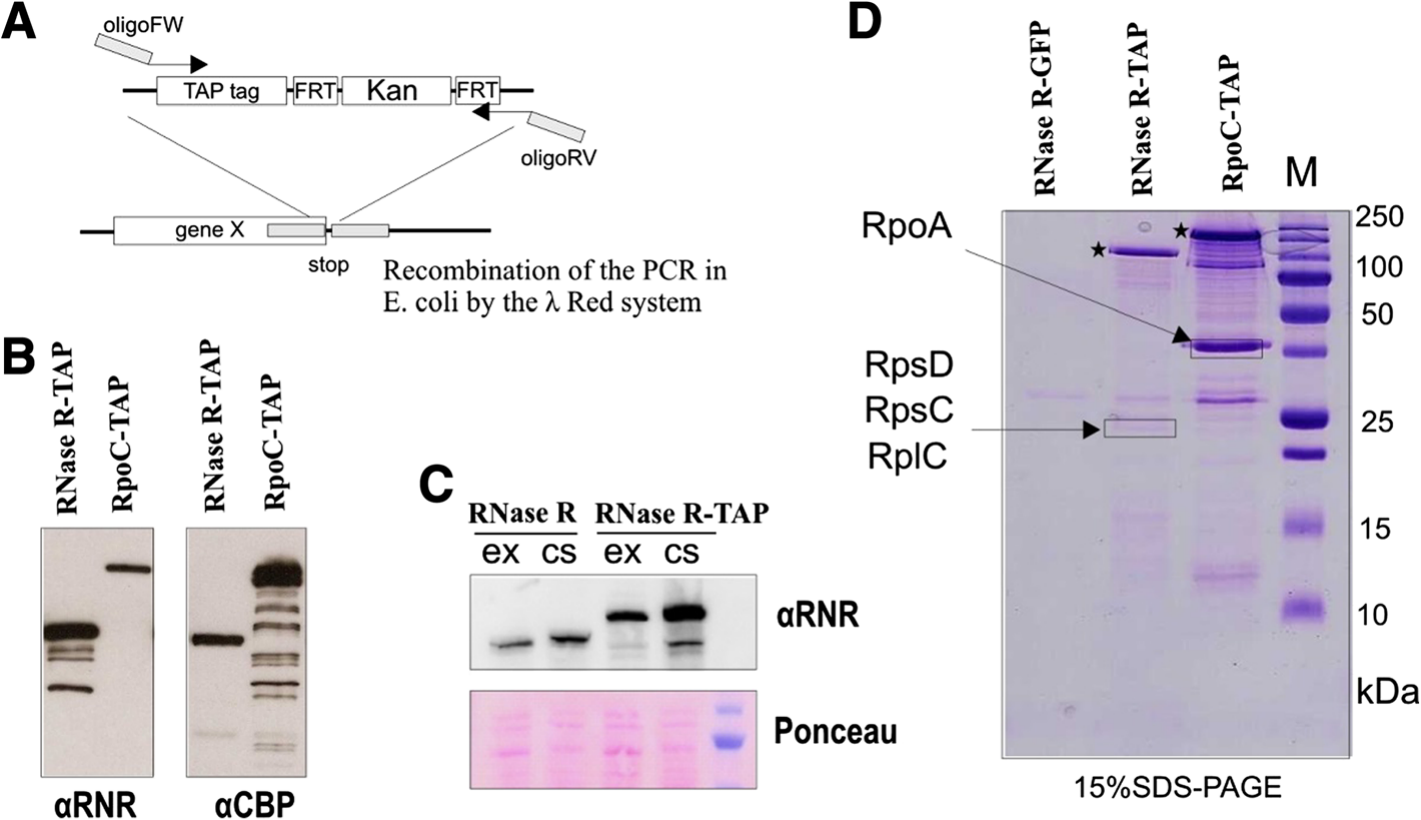
**Preparation of *E. coli *strains and TAP tag purification. (A)** Schematic representation of λ Red recombination strategy. PCR cassettes containing TAP tag sequence followed by kanamycin resistance gene (Kan) and flanked by FRT (flip recombinase targets) sites were prepared using primers with overhangs homologous to the sequences surrounding STOP codon of the chosen gene (gene X). After recombination TAP tag forms C-terminal translational fusion with the protein product of chosen gene. **(B)** Accuracy of the fusion proteins was monitored by western blot. Total bacterial proteins were subjected to western blot using α-RNase R antibodies (αRNR) or α- Calmodulin Binding Protein antibody (αCBP). Due to protein A in the TAP tag sequence the signal from RpoC-TAP fusion can be observed using α-RNase R antibodies. **(C)** Level of RNase R-TAP increases in a similar fashion as RNase R upon cold shock. Total bacterial proteins were subjected to western blot using α-RNase R (αRNR) antibody. Ponceau stain is provided as the loading control. ex- cells grown at 37°C until OD 0,5; cs- cells grown at 37°C until OD 0,5 and subsequently moved to 15°C for 4 h. **(D)** TAP tag purification of fusion proteins. Proteins from strains expressing RNase R-TAP, RpoC-TAP, or RNase R-GFP were purified [15], final elutions from calmodulin resin were separated on SDS-PAGE gel. Chosen bands (indicated by frames) were extracted and proteins were identified using mass spectrometry. Position of fusion proteins in the gel is indicated with stars.

We used the TAP tag purification method to obtain information about proteins interacting with RNase R *in vivo* (Figure 1A) [15]. The TAP tag sequence followed by a kanamycin resistance cassette was integrated into the *E. coli* genome to form a C-terminal translational fusion with RNase R protein [16]. A control strain with one of the RNA polymerase (RNAP) subunits - *rpo*C fused with a TAP tag was also constructed. Since RNAP is a well-defined protein complex, it served as a control for our purification method [17]. Additionally, we created a strain with RNase R protein fused with GFP that served as a negative control for TAP tag purification.

Chosen clones obtained after integrations of the cassettes were monitored by western blot to confirm the presence of the fusion proteins (Figure 1B). Additionally it was verified that C-terminal TAP tag fusion does not affect RNase R induction after temperature downshift (Figure 1C). The first purifications were performed according to the standard TAP tag procedures [15]. We detected sufficient amounts of target proteins in the final elutions in the case of both fusion proteins (Figure 1D).

Analysis of Coomassie-stained SDS-PAGE gels showed almost no background on the RNase R GFP fusion purification which proved specificity of the method used. In the case of the RpoC TAP fusion we saw enrichment on other RNAP subunits in the final elutions. One of the bands was extracted and mass spectrometry analysis proved that it corresponded to the RNAP subunit RpoA. In the RNase R-TAP fusion purification we mainly detected our target protein in the final elution, although there was some background enrichment compared to RNase R-GFP preparation. This result suggests that RNase R does not form stable complexes and that eventual interactions are rather transient. Similar results were obtained in several independent experiments using cells grown under different conditions (cold shock, exponential or stationary phase), and varying the amount of the background signal between the experiments (data not shown). Even though stable complexes formed by RNase R were not detected, some bands were found to be enriched in the RNase R-TAP preparation in relation to RNase R-GFP and RpoC-TAP. One of these bands was extracted from the gel and subjected to mass spectrometry analysis; which resulted in the detection of three ribosomal proteins: RpsD, RpsC and RplC (Figure 1D).

### RNase R does not form stable complexes but it does co-purify with ribosomal proteins

In order to obtain more comprehensive information about the transient interactions caused by RNase R we subjected the whole elution fraction to mass spectrometry analysis. For this analysis we chose the material obtained from cells subjected to cold shock treatment, since in this condition purification was the most efficient, probably due to increased levels of cellular RNase R [6]. We detected 212 proteins in the RNase R-TAP elution and 65 proteins in the control RpoC-TAP elution. Mass-spectrometry data were subsequently subjected to the label free quantification using MaxQuant software [18], which allowed relative values to be obtained that corresponded to the amount of each protein in the sample (intensity values).

In the graphical representation of the results the intensity values of the proteins identified in RNase R and RpoC samples were plotted against the specificity value of the protein in the samples. Specificity value corresponds to the ratio between the intensity of a given protein detected in the sample and the respective intensity of the protein in the control. Data obtained from RNase R-TAP purification were used as a control for the analysis of the data obtained from RpoC-TAP purification, and vice-versa.

**Figure 2:**
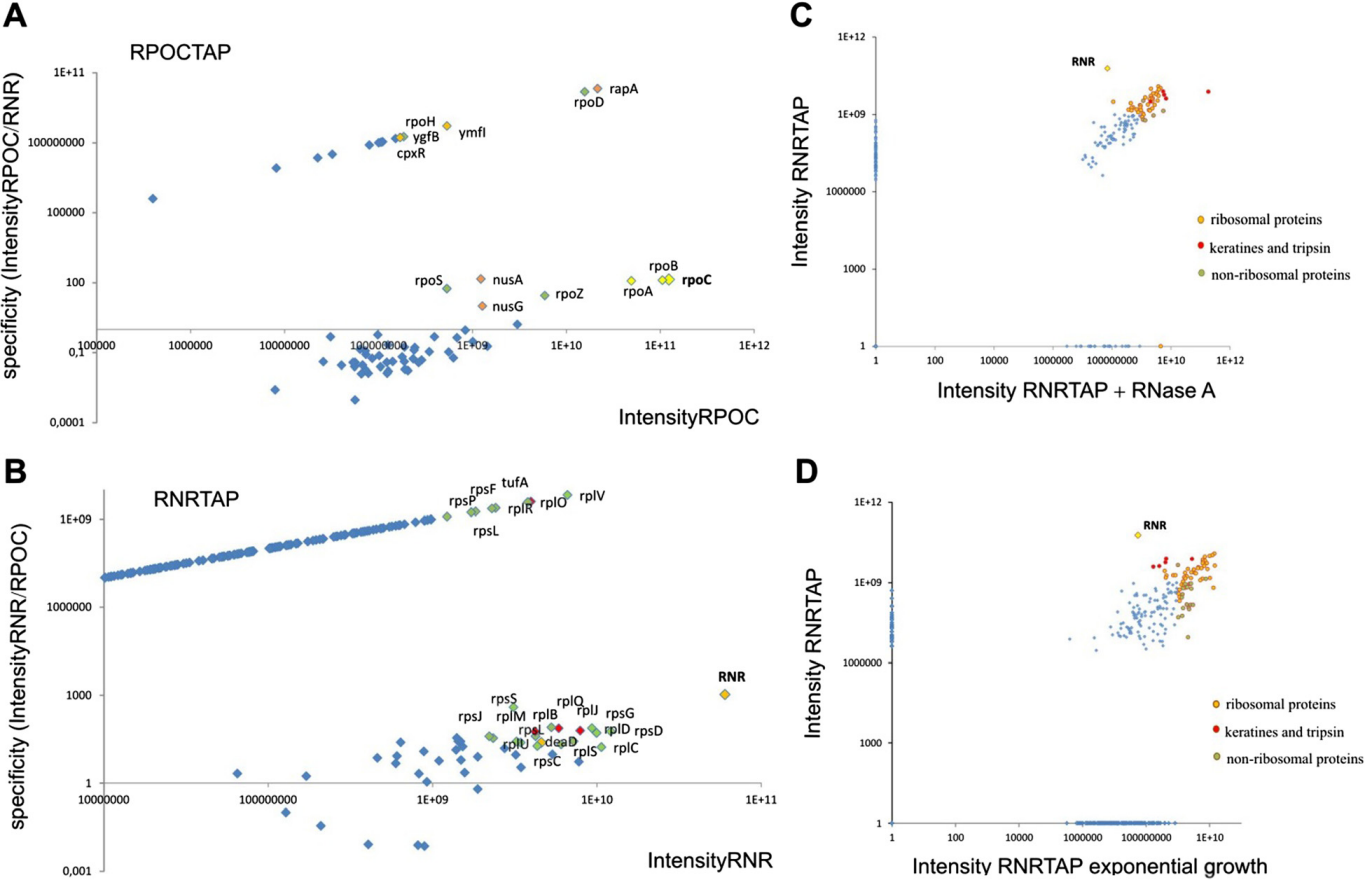
**Mass spectrometry analysis of TAP tag elutions.** Calmodulin elutions from RpoC-TAP or RNase R-TAP purifications were analyzed using mass spectrometry. Row data were subsequently treated by MaxQuant software for label free quantification of proteins amount in the sample (expressed as intensity value). In blue are represented the group of proteins that were detected with higher scores. **(A)** Proteins identified in RpoC-TAP sample. Intensity values of all proteins identified in calmodulin elution (x-axis) were plotted with specificity value of each protein (y-axis). Specificity is expressed as protein intensity value in the sample divided by intensity of given protein in the control sample. RNase R-TAP was the control sample for RpoC-TAP purification. **(B)** Proteins identified in RNase R-TAP sample. Intensity values of all proteins identified in calmodulin elution (x-axis) were plotted with specificity value of each protein (y-axis). RpoC-TAP was considered as control sample for RNase R-TAP purification. **(C)** Changes of protein content of RNase R-TAP elution sample in response to RNase A treatment. Intensity values of proteins detected in RNase R-TAP elution (RNRTAP) were plotted against intensities of proteins detected in RNase R-TAP sample from the experiment where RNase A was included into purification steps (RNRTAP + RNase A). Points with intensity values over threshold of 10^9^ are highlighted. **(D)** Changes of protein content of RNase R-TAP elution samples collected from exponentially growing cells compared to cells after cold shock (RNRTAP). Intensities of proteins detected in samples collected from the cells grown in different conditions were plotted. Points with intensity values over threshold of 10^9^ are highlighted.

Proteins detected with the highest intensity in RpoC TAP purification were all main RNA polymerase components (Figure 2A) [17]. The intensity values of the RNAP complex components were comparable to the value obtained for tagged protein RpoC, confirming that we could purify a stable RNA polymerase complex. A decrease of specificity for some of the complex components was due to their detection in the RNase R-TAP preparation. Interaction between RNase R and RNAP could not be ruled out under the chosen experimental settings. Apart from the five RNAP subunits, proteins more loosely connected with RNA polymerase were also detected, proving the sensitivity of the method. Interestingly, two proteins of unknown function, YgfB and YmfI, were detected with relatively high intensity values, suggesting that they may cooperate with the bacterial RNA polymerase complex (Figure 2A).

Proteins detected in the RNase R TAP sample generally had much lower intensity values compared to the tagged RNase R, going in agreement with the results observed with SDS-PAGE gels, and confirm the lack of stable complexes formed by RNase R (Figure 2B). Surprisingly, most of the proteins detected with relatively high intensities were ribosomal components. As in the case of RpoC-TAP, the specificity values of many proteins decreased due to its detection in the control sample. In order to check whether ribosomal proteins co-purified with RNase R due to an unspecific interaction provided by rRNA, we repeated the experiment adding RNase A during the purification steps. Results showed that after RNase A treatment the proteins detected with the highest intensities were still ribosomal components (Figure 2C). To check whether RNase R interaction with ribosomes was specific for cold shock, we performed mass spectrometry detection of proteins that co-purified with RNase R-TAP in exponentially growing cells. Comparison of the results showed that most of the proteins detected were the same under both conditions (Figure 2D). This suggests that interaction between RNase R and ribosomes is not an artifact of the growth conditions.

There was a drop in the intensity value of RNase R obtained by mass spectrometry between RNase R TAP sample after RNase A treatment and the sample from exponentially growing cells. We consider it as a method artifact since this effect did not reflect the amount of RNase R in the sample estimated by SDS-page gels (data not shown).

### RNase R interacts mostly with non-translating ribosomes *in vivo*

Analysis of the mass spectrometry data suggested that there can be physical interaction between RNase R and the ribosomes. To explore this we used sucrose polysome gradients and detected the RNase R position in the gradient using antibodies against RNase R. During centrifugation of total bacterial extracts in sucrose gradients, the soluble proteins stay at the top, whereas ribosomes migrate deeper into the gradient due to their size. The relation between the position of RNase R and ribosomes along the gradient should reveal eventual interactions between these two particles. The use of anti RNase R antibodies to detect the RNase R position in the gradient enables the observation of the behaviour of the endogenous untagged proteins.

**Figure 3:**
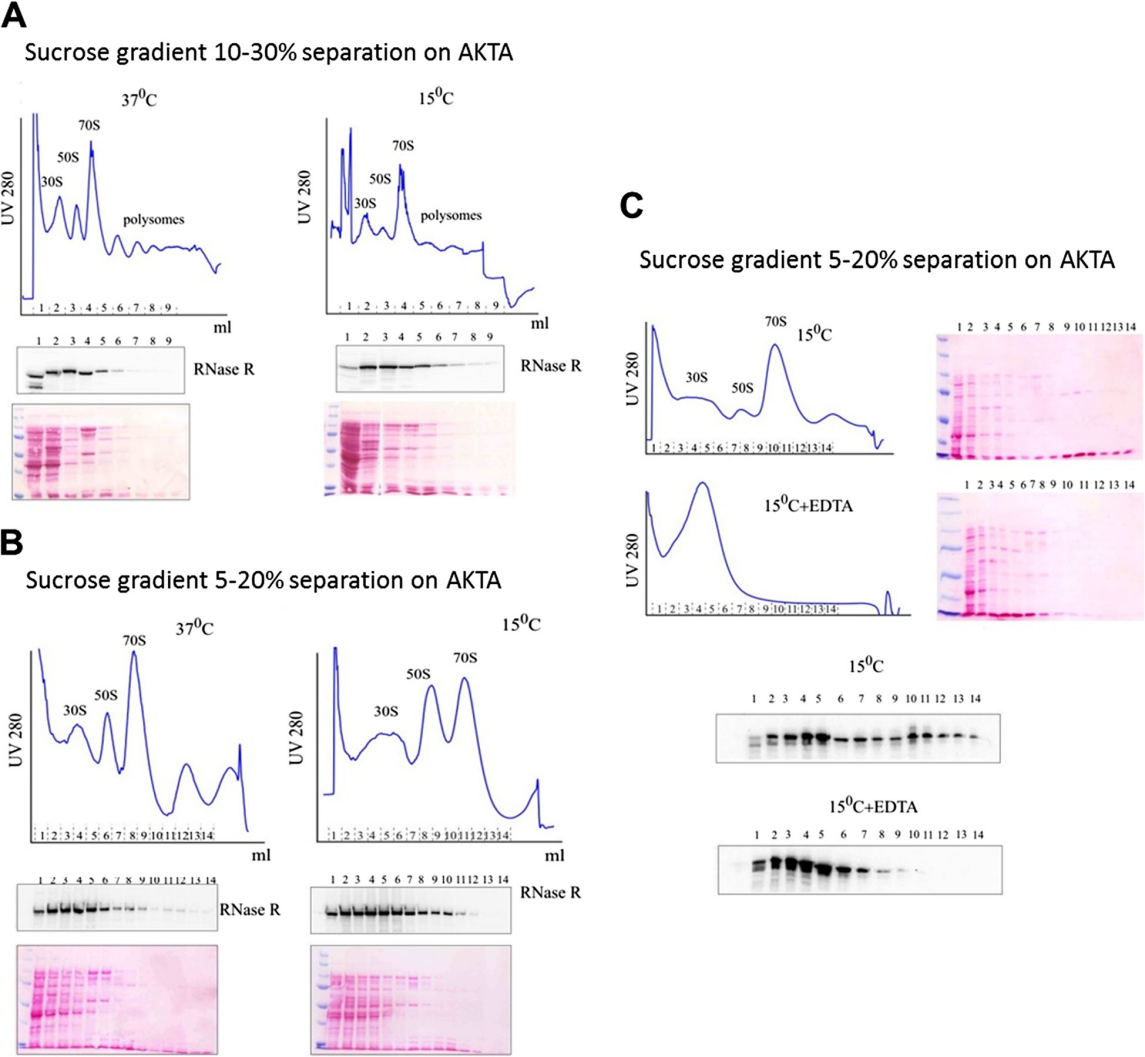
**RNase R interacts with the small ribosomal subunit.** Cellular extracts were separated on sucrose gradients. Position of ribosomal subunits, ribosomes and polysomes along the gradient were monitored by UV 280 absorbance (UV280). Amount of RNase R in each fraction of the gradient was monitored using western blot. Amount of proteins along the gradient was monitored by Ponceau stain. **(A)** 10-30% sucrose gradient. Polysomes were separated from exponentially and cold shocked cells. **(B)** 5-20% sucrose gradients. Polysomes were separated from exponentially and cold shocked cells. Difference in subunits migration between the gradients is due to longer centrifugation time of cold shock sample. **(C)** 5-20% sucrose gradients. Polysome from cold shocked cells were separated, part of the sample was treated with EDTA which results in ribosomal subunits separation. The treatment changes pattern of RNase R in the gradient indicating its interaction with ribosomes.

Western blot analysis of the gradient fractions showed that the RNase R signal reached maximal intensity not at the top of the gradient, as expected for soluble proteins, but a few fractions deeper (Figure 3A). Similar results were obtained for the cells grown at 37°C and the cells after the cold shock treatment; although cold shock treated cells gave a stronger signal due to the increase in the RNase R level. As a control we have used RNase II, a protein from the same family. In contrary to RNase R, RNase II does not migrate along the sucrose gradient. This protein remains mostly in the fraction of the gradient corresponding to the soluble proteins, showing no interaction with the ribosomes (see Additional file 2: Figure S1). If we compare the signal intensity and position of the ribosomal subunits using less dense gradients, we can observe that the RNase R enrichment corresponds mostly to the single 30S ribosomal subunit. Interestingly, interaction between RNase R and the small ribosomal subunit protein S12, encoded by the *rpsL* gene, has recently been proposed, leading credence to our conclusions [19]. After reaching its maximum, RNase R signal intensity decreased along the gradient, but it could still be detected in the fraction corresponding to the 50S subunit and until the peak of the 70S ribosome (Figure 3A,B). The weaker detection of RNase R in the 50S subunit can be explained by the interaction of this enzyme with DeaD (also known as CsdA). DeaD is a helicase involved in the biogenesis of the 50S ribosomal subunit and its deletion leads to the dysfunction in biogenesis of this ribosomal subunit [20].

Sample treatment with EDTA, which results in ribosome disruption and subunit separation, causes a change in the RNase R signal pattern, indicating that the position of RNase R in the gradient was due to an interaction with the ribosomes (Figure 3C).

### RNase R deletion does not impact ribosome formation

Our results show that RNase R *in vivo* interacts with the ribosomes. Data from independent studies suggest that RNase R is involved in the ribosome quality control [9,10], so interaction with the ribosomes can be important for this function. Overexpression of RNase R rescues phenotype of DeaD helicase deletion at low temperatures. One of the phenotypes of DeaD deletion is the dysfunction in biogenesis of 50S ribosomal subunit [5,21]. The suppressing role of RNase R suggests that it may also be involved in the ribosome biogenesis.

**Figure 4:**
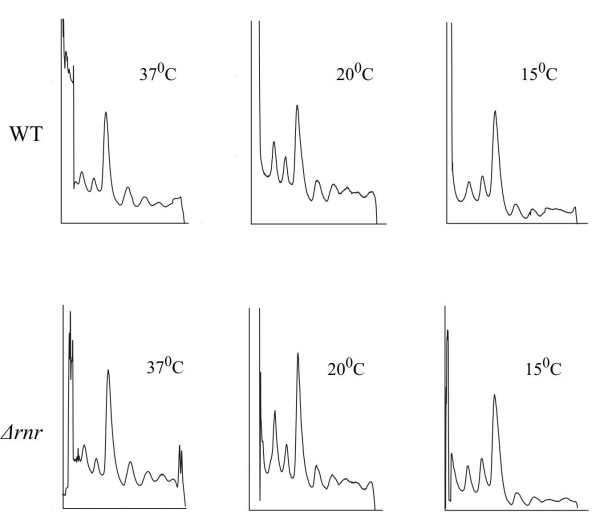
**RNase R deletion does not impact polysome profiles.** Cellular extracts from RNase R deletion cells and wild type cells were separated on sucrose gradients. Samples were collected from the cells grown at different temperatures: 37°C 20°C and after cold shock (37°C followed by 4 h at 15°C).

If RNase R is important for ribosome biogenesis, deletion of this enzyme may cause changes in ribosome number or accumulation of deficient ribosome species. To check such a possibility, the sucrose polysome profile of an RNase R deletion strain was compared to those obtained with the wild type cells. Polysome samples were analyzed from the cells growing under different temperatures: 37°C, 20°C and after four hours of cold shock treatment (15°C). We did not noticed significant difference in polysome profiles between wild type and RNase R deleted strain in none of the conditions tested (Figure 4). The relative amount of whole ribosomes and the single subunits were comparable, as well as the amount of polysomes that reflect the conditions of the translation machinery. Also, no accumulation of new dysfunctional ribosome species was observed. We did not detect any significant difference after a prolonged incubation of the cells at low temperature (data not shown). This suggests that RNase R function in ribosome biogenesis is redundant and can be executed by other enzymes under its absence.

## Discussion

In this study we investigated potential interactors of *E. coli* RNase R using TAP tag purification in combination with mass spectrometry protein identification. Our results suggest that RNase R does not form stable complexes *in vivo*, but it can interact with ribosomal proteins. Surprisingly, among the proteins that co-purify with RNase R we did not detect any components of the trans-translation pathway, although interaction of RNase R with SsrA and SmpB complex was previously detected using SmpB immunoprecipitation [13]. During trans-translation, RNase R is recruited to stalled ribosomes by an interaction of its C- terminal region with the components of the trans- translation machinery [22]. Because in our experiments we used a C-terminal TAP tag fusion, part of the interactions in this protein region could have been lost.

The detected interaction of RNase R with the ribosomes was supported by the analysis of sucrose polysome gradients with antibodies against RNase R. Endogenous RNase R migrates in the sucrose gradients in a similar fashion as the 30S ribosomal subunit. Moreover, treatment of the sample with EDTA changed the RNase R migration pattern. Previous studies suggested an interaction between RNase R and the 30S ribosomal protein S12, which is in agreement with our observations [19].

Although our work proves an interaction between ribosomes and RNase R, we did not detect any difference in the ribosome profiles after *rnr* gene deletion. This suggests that whatever is the biological function of RNase R connected to the ribosomes it is redundant, and can be executed by other enzymes. Redundancy of exonucleases functions is common in *E. coli* and deletion of any of the three main exonucleases has any or minimal, effect on the cell fitness [23].

**Figure 5:**
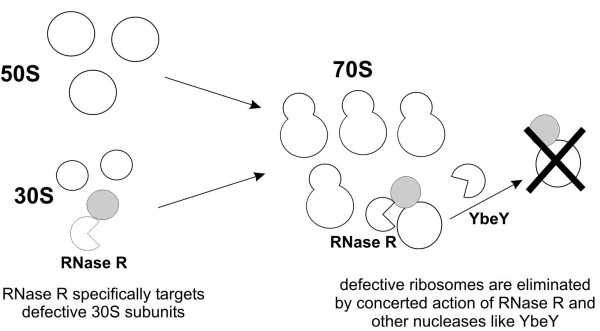
Hypothetical model of RNase R involvement in tagging and removing defective ribosomes.

Based on the results provided by other independent study we believe that the main function of RNase R connection with the ribosomes is its involvement in ribosome quality control. Recently, it was shown that RNase R together with YbeY nuclease can efficiently degrade deficient ribosomes *in vitro*, and this function is dependent on the presence of both enzymes [10]. RNase R and YbeY can only degrade complete 70S ribosomes, but not single subunits [10]. Although we observed that most of cellular RNase R signal co-migrates with the ribosomes at the sucrose gradient, it does not mean that all cellular ribosomes are linked with RNase R. Based on approximate estimations of protein copy numbers in the cell, we can predict that in exponentially growing cells ribosomes are at least 100 fold more abundant than RNase R, which means that RNase R is only connected to a small fraction of the cellular ribosomes [24]. We are tempted to speculate that RNase R can specifically target deficient ribosomal 30S subunits, which subsequently results in the specific 70S ribosome degradation by YbeY and RNase R. This explains why we could see a specific enrichment of RNase R on 30S subunit but not on the 70S ribosome, which would be rapidly degraded (Figure 5). We aim to explore this hypothesis in our future work.

## Conclusion

In conclusion, this study shows that RNase R can interact with ribosomal proteins. Using sucrose polysome gradients combined with anti-RNase R antibodies we showed that endogenous RNase R migrates along the gradient in a similar fashion as the 30S ribosomal subunit. RNase R is usually more abundant in the 30S fraction and this result is coherent with previous data since it was reported that it associates with the S12 protein [19]. However, in the westerns we see that RNase R can be associated with the other two subunits, 50 and 70S. This protein is not visible on the polysome fraction but it can be associated with 70S.

## Methods

BW25113 *E. coli* strain was used in all described experiments. Bacteria were grown in standard LB media. All cultures were incubated under aerobic conditions at 37°C and shaken at 180 rpm. Strains with fusion proteins were prepared using lambda red recombination method as described [16]. Plasmids were used as a template for the integration cassettes as described [25].

### TAP tag purification

Tap tag purification was performed as described [15]. *E. coli* with RNase R protein fused with TAP tag cultures were grown in LB medium with kanamycin (50 μg/ml) until they reach the exponential or the stationary growth phase. Cold shock induction cultures that reach exponential phase were incubated for 3 h at 15°C. Cells were harvested and pellets stored at -80°C. Pellets were resuspended in 8 ml of Lysis buffer (2 mM PMSF, 1 mM DTT, 50 mM Tris-HCl pH8.0, 250 mM NaCl) and lysed by two passages in French press. At this point 0.5 μl of benzonase (250 U/μl) was added and samples were incubated on ice for 10 minutes and centrifuged at 35000 rpm for 45 minutes at 4°C. Supernatants were filtered (0.45 μm), supplemented with 0,1% Triton ×-100 and loaded into pre-washed IgG agarose column. The columns remained shaking at 4°C for 1.5 h, then were washed twice with 10 ml of IPP150 and subsequently two times with 10 ml of TEV Cleavage Buffer (10 mM Tris-HCl pH8.0, 150 mM NaCl, 0.5 mM EDTA, 1 mM DTT). A volume of 200 μL of TEV Cleavage Buffer and 35 μL of TEV protease (approximately 100 units) were added to the column and incubated for 1 h shaking at room temperature. After that, the supernatant was collected by eluting twice with 250 μL of Calmoduline Binding Buffer (CBB) (10 mM β-mercaptoethanol, 10 mM Tris-HCl pH 8.0, 150 mM NaCl, 0,1% Triton X-100, 2 mM CaCl2). The sample was supplemented with CaCl_2_ (4 μl of a 0.2 M stock) and the mixture was transferred into an eppendorf with 300 μL of Calmoduline beads (previously washed 4 times with CBB). Incubation was performed for 45 minutes while shaking at 4°C; subsequently the sample was transferred into a new column and washed twice with 5 ml of CBB. As a final step, we eluted proteins with 600 μL of Calmoduline Elution Buffer (10 mM β-mercaptoethanol, 10 mM Tris-HCl pH 8.0, 150 mM NaCl, 1 mM CaCl2). If indicated 0.1 μg/ul of RNase A was added during the step of binding to calmodulin resin. Final elutions were precipitated with acetone and pellets were sent for mass spectrometry service (http://mslab-ibb.pl/). Raw mass spectrometry data were treated using MaxQuant software to obtain label free quantification data [18].

### Sucrose gradient separation

Sucrose gradients were prepared as described [5]. Cultures were grown until the exponential growth phase and/or in cold shock (as described before). Chloramphenicol was added into the culture (final concentration 0.1 mg/ml) which remained for 3 minutes shaking under the same conditions. Cultures were transferred into a centrifuge tube filled until 1/3 of the volume with ice, centrifuged at 5000 rpm, during 10 minutes at 4°C. Pellets were resuspended with 0.5 ml of cold Buffer A (100 mM NH_4_Cl, 10 mM MgCl_2_, 20 mM Tris-HCl pH 7.5), transferred into an eppendorf and lysozyme solution was added to a final concentration of 0.1 ug/ul. Cells were frozen in liquid nitrogen for 5 minutes and then thawed in an ice water bath (this step was repeated twice). Subsequently, 15 ul of 10% Deoxycholate was added to complete the cell lysis and the sample was centrifuged at 17000 rpm for 10 minutes at 4°C. Supernatant was carefully transferred into a new eppendorf and stored at -80°C. Amounts of RNA were determined using NanoDrop equipment and approximately 600 ug was added into the top of the sucrose gradient. Samples were centrifuged at 35000 rpm for 3 h at 4°C, using an SW41 Ti rotor. Gradients were separated using AKTA equipment and UV spectra were monitored. Gradient fractions were precipitated with TCA and proteins were separated on SDS-PAGE gels and subjected to standard western blot analysis. If indicated 40 mM EDTA was added to the lysates before separation on the gradient.

## Competing interests

The authors declare that they have no competing interests.

## Authors’ contributions

MM and BC had equal contribution. All authors read and approved the final manuscript.

## Supplementary Material

Additional file 1: Figure S1 RNase R interacts with the small ribosomal subunit. Cellular extracts were separated on 5-20% sucrose gradients. Position of ribosomal subunits, ribosomes and polysomes along the gradient were monitored by UV 280 absorbance (UV280). Amount of RNase R or RNase II (used as a control) in each fraction of the gradient was monitored using western blot. Click here for file

Additional file 2: Table S1 Mass Spectrometry results from TAP tag purification. List of proteins co-purified with RNase R or RpoC during cold shock induction, in exponential growth phase and after RNase A treatment. Click here for file
