# Supplementary material for: Characterization of the RNase R association with ribosomes
Source: BMC Microbiol. 2014 Feb 11;14:34. doi: 10.1186/1471-2180-14-34 (PMC3942186; doi:10.1186/1471-2180-14-34)
Supplement: Additional file 2: Table S1 — Mass Spectrometry results from TAP tag purification. List of proteins co-purified with RNase R or RpoC during cold shock induction, in exponential growth phase and after RNase A treatment. [file 1471-2180-14-34-S2.pdf]

Supplementary data

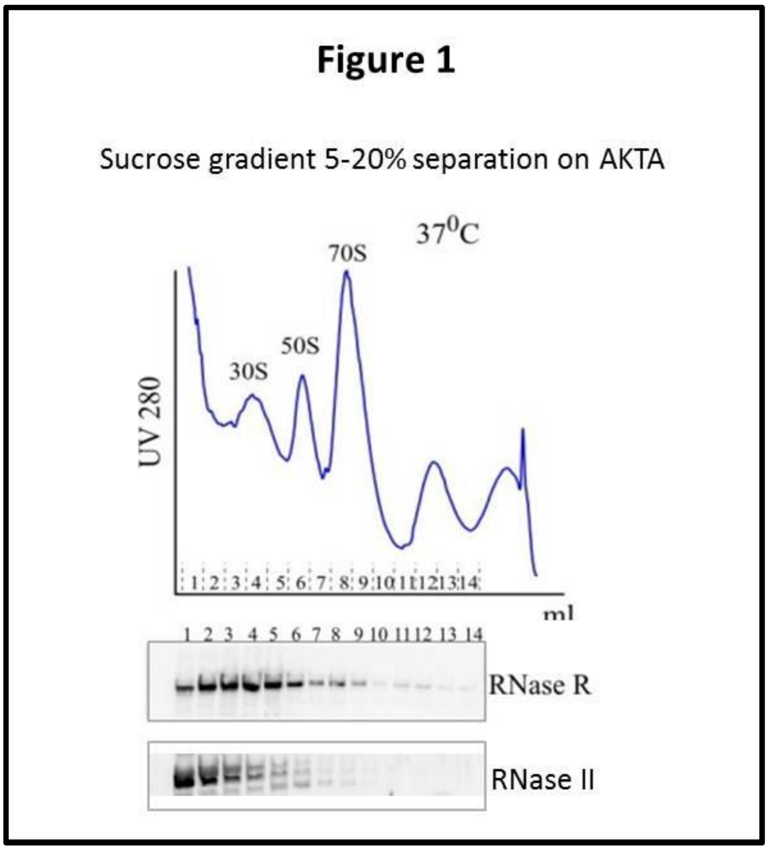

Fig.1. RNase R interacts with the small ribosomal subunit. Cellular extracts were separated on 5-20% sucrose gradients. Position of ribosomal subunits, ribosomes and polysomes along the gradient were monitored by UV 280 absorbance (UV280). Amount of RNase R or RNase II (used as a control) in each fraction of the gradient was monitored using western blot.
